# Efficient noble metal promoted bimetallic cobalt catalysts in the selective synthesis of acetaldehyde dimethyl acetal†

**DOI:** 10.1039/d3ra02784h

**Published:** 2023-07-26

**Authors:** Kalim A. Sheikh, Thomas A. Zevaco, Jelena Jelic, Felix Studt, Michael Bender

**Affiliations:** a Karlsruher Institut für Technologie (KIT) Eggenstein-Leopoldshafen Germany kalim.sheikh@kit.edu thomas.zevaco@kit.edu; b BASF SE Ludwigshafen Germany

## Abstract

Herein we report the one-pot cobalt catalysed synthesis of the dimethylacetal of acetaldehyde from synthesis gas and methanol. The product can be used as a fuel additive either as it is or after transacetalisation with long-chain alcohols. The product is obtained at moderate temperatures in good selectivities and high CO-conversions. A variation of the promotor metal (Au, Pt, Pd, and Ru) and of the support (γ-Al_2_O_3_ and CeO_2_) in the catalyst was conducted, which showed a great impact of both the support and promotor on the activity and structure of the catalyst. Furthermore, a specific variation of temperatures and pressure for the most active catalyst and a model catalyst was conducted giving an interesting insight into ongoing processes.

## Abstract 2

Herein we report the one-pot cobalt catalysed synthesis of the dimethylacetal of acetaldehyde from synthesis gas and methanol.

## Graphical abstract

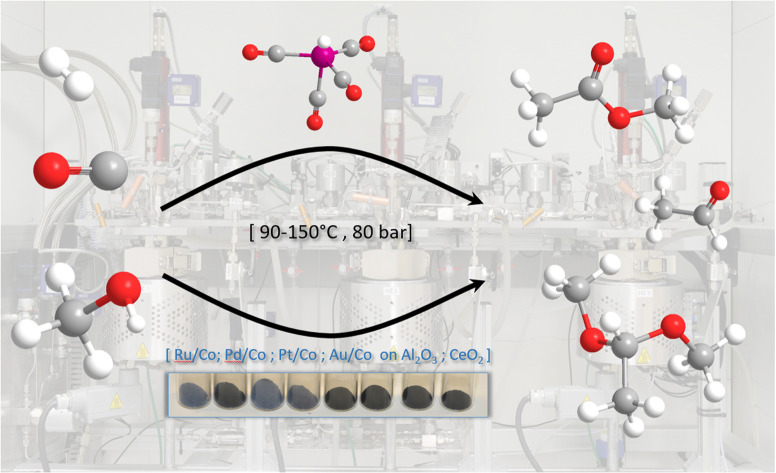

## Introduction

Acetaldehyde dimethyl acetal (AADMA) is a promising substance for the synthesis of many kinds of pharmaceuticals and fragrances and can be also efficiently used, such as the familiar dimethoxymethane, as a diesel additive to enhance the cetane number.^1^ Structurally very similar trialkoxyalkanes also reduce soot emissions, as known for most oxygenates.^1–3^ It is not only used as a raw material but is also an important intermediate species for the synthesis of various oxygen-containing organic materials, such as higher acetals, resins, solvents, and adhesives. The direct synthesis from CO, H_2,_ and methanol was investigated rather sporadically. The first publications in this field focused on the chain prolongation of alcohols, the so-called homologation that was revealed to be a tedious procedure due to a complex separation of the products. Wender *et al.* in 1949 reported the successful homologation of methanol to ethanol in good yields.^4,5^ They reacted methanol with carbon monoxide and hydrogen in the presence of a cobalt catalyst.^6^ Before discovering this reaction, synthesizing longer chained alcohols was a multistep procedure starting from the Fischer–Tropsch reaction typically yielding C_*n*_-olefins able to be hydroformylated to the corresponding C_*n*+1_-aldehydes and then reduced to the related alcohols, which are of great value as surfactants. Wender *et al.* showed that using benzyl alcohol, an alcohol not able to form an olefin, the cobalt-catalyzed homologation reaction did not proceed by the hydroformylation mechanism.^4^ W. Reppe, a pioneer in high-pressure chemistry, first patented the direct synthesis of AADMA. In 1949, Reppe *et al.* patented the process for the synthesis of “oxygen-containing organic compounds”. They stated that with the use of cobalt bromide or cobalt iodide at pressures up to 650 atm it was possible to achieve high methanol conversions and selectivities towards AADMA of up to 77%.^7^ The long residence time of 50 h and the separation of the crude mixture were the major drawbacks of this process. In 1955, Reppe *et al.* proceeded by adding quaternary alkyl ammonium or phosphonium halides to the mixture of methanol and CoX_2_ (X = chloride or bromide) salts. This procedure generates *in situ* the active tetrabromo- or tetraiodo cobaltate complexes. They noticed that the addition of pyridinium cations improved the selectivities towards AADMA but lowered the overall methanol conversion. Whereas, triethylbutylammonium cations increased the selectivity towards methylacetate (MeOAc), phosphonium cations led to an increased selectivity towards acetic acid and MeOAc and a low AADMA selectivity.^8^ Further improvements in this field showed that the use of nickel as co-catalyst lowers residence times and gives conversions of over 50% in 1 h.^9^

Most of the publications are limited to the homogenous process for the synthesis of AADMA.^10,11^ Reports on the heterogeneously catalysed synthesis of AADMA from methanol and synthesis gas are scarce. Recently, Blair's group published a gas phase synthesis of acetaldehyde on gold particles on a single layer of MoS_2_ supported on silica. They report a gas phase reaction of methanol and CO at mild conditions of around 308 kPa and 393 K towards the critical intermediate acetaldehyde. The experimental results were validated with density functional theory calculations, which proposed a viable reaction pathway. Scanning electron microscopy showed that MoS_2_ enhanced the formation of Au-rich aggregates on the surface of the catalysts during the synthesis, which played a vital role in the catalytic activity.^12^

**Scheme 1:**
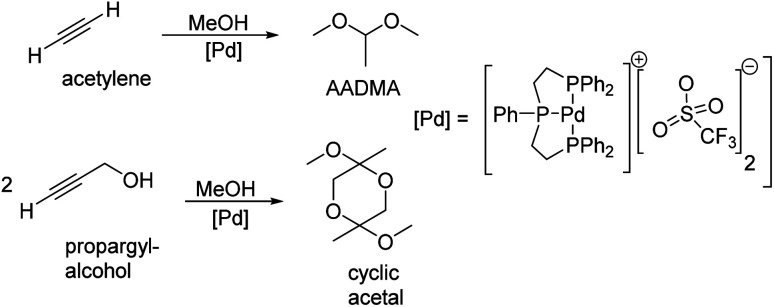
Peringer *et al.* described the synthesis of dimethylacetals starting from alkynes and alkynols.^13^

Peringer *et al.* published in 1996 a more exotic synthesis route to AADMA and other dimethyl acetals starting from a palladium complex. They showed that by using tridentate ligand, as shown in Scheme 1, a general synthesis of dimethylacetals from alkynes can be conducted in the liquid phase. They also noticed, that using alkynols, such as propargyl alcohol, gives access to cyclic acetals.^13^

A third way to obtain AADMA is *via* acetalisation of a solution of methanol and acetaldehyde. Acetaldehyde is a highly reactive electrophile, which makes it susceptible to nucleophilic attack on the carbonyl group. Studies on the reaction of acetaldehyde with methanol were performed in 1952 by Darwent and Meadows.^14^ They investigated the nature of the products obtained from the reaction of acetaldehyde with methanol in neutral and acidic solutions. They noted that at low temperatures hydrochloric acid does not catalyse the formation of aldols or of oligomeric/polymeric products and distinguished only AADMA as the sole product.^14^ This process compared to the processes described by Reppe *et al.* gives the advantage that no other products such as methyl acetate, acetic acid, or ethanol were observed, which makes the work-up of the reaction easier. Smith Jr. *et al.* patented this procedure as a general approach to acetals using an acid-catalyzed reaction of an alcohol with an aldehyde.^15^ A thermodynamic and kinetic investigation of this reaction was performed by Rodrigues *et al.* in 2005. They used the acidic resin Amberlyst-15 and a Y-type zeolite as the catalysts and compared the results. They could experimentally assess reaction constants such as the equilibrium constant in the range of 20–60 °C as well as, Gibbs free energy, enthalpy, and entropy.^16^

We herein report a study on the synthesis of acetaldehyde dimethyl acetal (AADMA) and side products such as methyl acetate (MeOAc) or methyl formate (MeFo). Structurally, very similar formaldehyde dimethylacetal (DMM) is a highly attractive molecule in the context of synthetic fuels and fuel additives for soot reduction during combustion. Many studies have placed the focus on an efficient one-step synthesis route towards DMM using methanol and/or synthesis gas.^17^ However, only low conversions due to thermodynamic limitations of the reductive route could be reached. In comparison, AADMA could be synthesised from the desired reactants, methanol and synthesis gas, in the liquid phase at much higher yields and rates. The first results will be presented in this contribution where interesting aspects of the reaction mechanism could also be understood.

## Results and discussion

The supported cobalt catalysts used in this study were synthesised by a wet impregnation method. The catalysts were optimised with different noble metal promotors such as Au, Pt, Pd, and Ru. Supports γ-Al_2_O_3_ and CeO_2_ were used and impregnated with the noble metal precursor and the cobalt precursor simultaneously. After impregnation and drying, the catalysts were calcined and reduced. The reduced and calcined catalysts were characterised by X-ray fluorescence (XRF), inductively coupled plasma coupled with optical emission spectroscopy (ICP-OES), powder X-ray diffraction (PXRD), and scanning electron microscopy (SEM).

### Catalyst characterisation

The results for the XRF and ICP-OES measurements after synthesis of the catalysts are shown in Table 1. Small differences between XRF and ICP methods for determination of metal contents on supported catalysts were noted, keeping in mind that both methods inherently differ in sample preparation and measuring techniques (for details see the Experimental section). Greater deviations from the desired 1 wt% of noble metal loading could be seen for the Ru-promoted catalysts. Ruthenium is known to build volatile oxides, which can leave the catalyst surface during the calcination and therefore reduce the actual loading.^18,19^

**Table 1:** Metal loadings measured with XRF and ICP-OES method for the synthesised X/Co catalysts supported either on γ-Al_2_O_3_ or CeO_2_

Catalysts	X-loading (wt%)		Co-loading (wt%)	
	XRF	ICP	XRF	ICP
Co/Al_2_O_3_	—	—	11.6	9.6
Ru/Co/Al_2_O_3_	0.34	0.33	9.4	9.4
Pd/Co/Al_2_O_3_	0.57	0.89	12.4	11.2
Pt/Co/Al_2_O_3_	1.12	0.93	11.5	8.9
Au/Co/Al_2_O_3_	0.73	0.80	11.5	9.0
Ru/Co/CeO_2_	0.56	0.61	9.8	9.2
Pd/Co/CeO_2_	0.80	0.86	10.4	9.6
Pt/Co/CeO_2_	0.97	0.97	11.0	9.1
Au/Co/CeO_2_	0.77	0.96	9.3	10.2

**Fig. 1:**
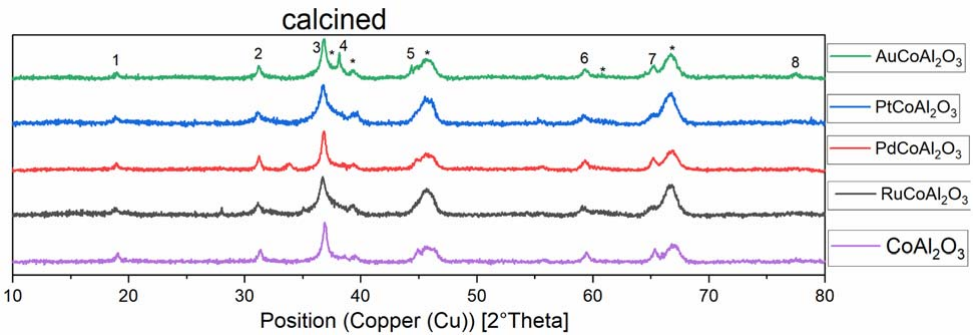
Normalised XRD diffractograms of all catalysts supported on γ-Al_2_O_3_ measured at the calcined (oxidic) state. Numbers show the peaks for Co_2_O_3._ * = γ-Al_2_O_3_ ∼ = Au.

**Fig. 2:**
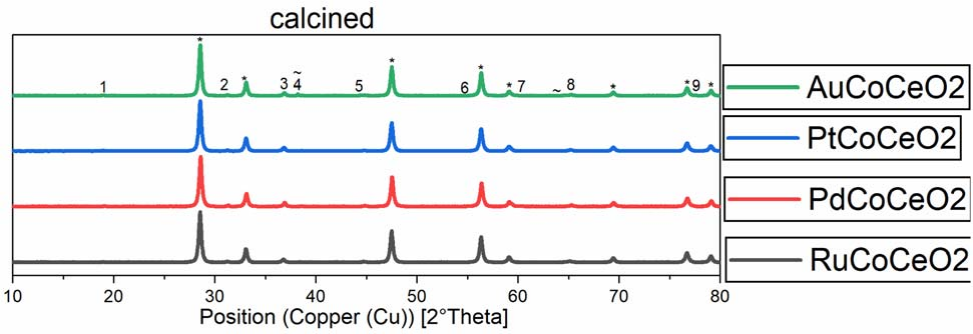
Normalised XRD diffractograms of all catalysts supported on CeO_2_ measured at the calcined (oxidic) state. Numbers show the peaks for Co_2_O_3_. * = or CeO_2_ peaks, ∼ = Au peaks.

XRD diffractograms of all calcined samples are shown in Fig. 2, exemplarily, the Au-promoted cobalt catalyst samples are highlighted for both supports γ-Al_2_O_3_ (top) and CeO_2_ (bottom) in the calcined state. The comparison of the peaks of the supports clearly shows the difference in crystallinity. The nanocrystals of CeO_2_ showed a high amount of crystallinity compared to the amorphous γ-Al_2_O_3_, which led to broad peaks. A strong dependency on the metallic cobalt peak intensity of the reduced state catalysts with varying promoters could be noticed. This is a hint towards the reducibility of cobalt oxides on the surface of the catalyst, which is increased in the presence of gold and platinum precursors (see Fig. 1 and 2).

After fitting the XRD peaks for the (311)-Co_3_O_4_- and the (220)-Co_3_O_4_-peak for the calcined catalysts, the values for the crystallite size could be evaluated. An average cobalt crystallite size was calculated from Scherrer's equation, this method is common to quickly assess crystallite size.^20,21^ The calculated values are summarised in Table 2. For the γ-Al_2_O_3_-supported catalysts a decrease in particle size is observed by co-impregnating cobalt with Ru, Pt, and Au. In comparison, the co-impregnation of palladium leads to an increase in particle size compared to the non-promoted catalyst Co/Al_2_O_3_. For the CeO_2_-supported catalysts, the calculated particle size was generally bigger, which can be tentatively explained by stronger metal support interactions. Again, the largest particle size was observed for the Pd-promoted cobalt on the CeO_2_ catalyst (see Table 2). Generally, the values are in good agreement with the literature.^22–24^

**Table 2:** Calculated crystallite sizes from fitted XRD diffractograms for Co^0^ from Scherrer's equation

Catalysts	*d*(Co^0^) [nm] from calcined state	
	(311)-phase	(220)-phase
Co/Al_2_O_3_	14	20
Ru/Co/Al_2_O_3_	7	6
Pd/Co/Al_2_O_3_	22	26
Pt/Co/Al_2_O_3_	7	7
Au/Co/Al_2_O_3_	9	11
Ru/Co/CeO_2_	45	82
Pd/Co/CeO_2_	72	92
Pt/Co/CeO_2_	42	69
Au/Co/CeO_2_	37	14

### Catalyst testing

#### Screening of different cobalt on alumina catalysts and temperature effects

**Scheme 2:**
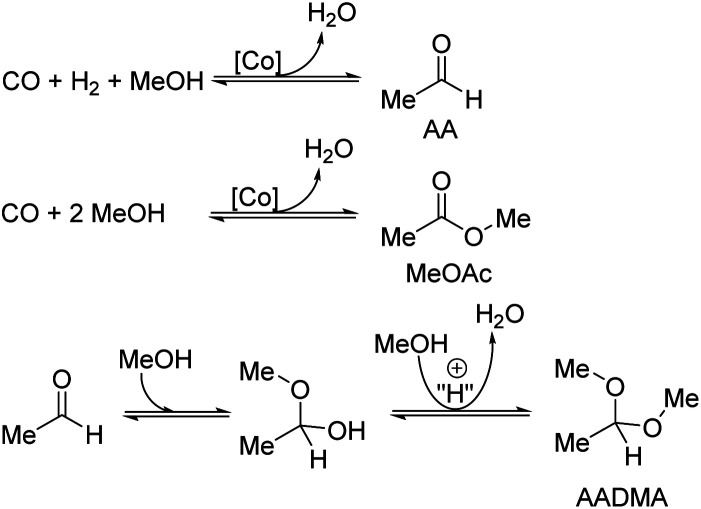
Overview of major ongoing reactions.

All shown catalysts were investigated under mild conditions to prevent unwanted side reactions of acetals, esters, and carbon monoxide. Most commonly methanisation, polymerisations, aldol reactions, and Fischer–Tropsch reactions are the side reactions that can be catalysed by cobalt at higher temperatures (>150 °C). The major ongoing reactions are cobalt- and acid catalysed reactions (see Scheme 2).

**Scheme 3:**
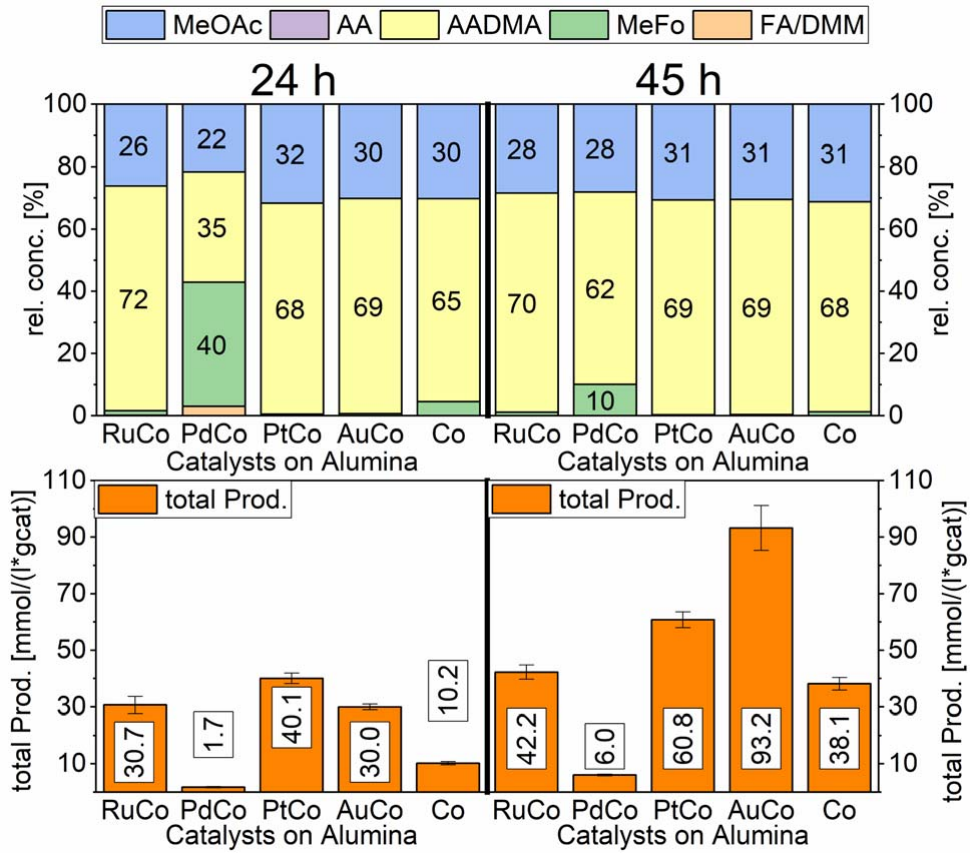
Comparison of total productivity and relative concentrations of Co/Al_2_O_3_ co-impregnated with different promoter metals. Testing conditions: 80 bar, 90 °C, CO : H_2_ 1 : 3, 50 mL methanol, quantification: GC-FID (PolyArc), 1 g of the catalyst.

A strong activity dependency with the reaction time and nature of the noble metal promoter was observed (see Scheme 2). The strong increase in product concentrations between 24 and 45 h strongly suggests, that the reaction is in a kinetic regime and even after 45 h not in an equilibrium state. A difference in the catalytic activity between noble-metal-containing powders and pure cobalt on γ-Al_2_O_3_ can be clearly seen, with the least active catalyst being the one promoted with palladium. Even after 45 h of operation, the productivity is negligible. The Ru-promoted catalyst showed similar activity to the catalyst with no promoter. Pt- and Au-promoted catalysts showed the highest activity of all investigated catalysts (see Scheme 3). According to the literature, Pt and Au are known to be reduction promoters of several cobalt oxide species.^25^ By the so-called hydrogen spillover effect, these elements increase the reducibility and therefore the amount of active metallic cobalt on the surface of alumina.^26^ This behaviour will be later explained by the help of DFT-calculations.

According to many studies, cobalt is known to leach easily from alumina surfaces in the presence of CO.^27^ In the liquid phase leaching is enhanced so that an equilibrium state between cobalt in the solution and cobalt on the surface of the support is reached. High partial pressures of CO led to the formation of cobalt carbonyl species, mainly Co_2_(CO)_8_.^28^ The cobalt carbonyl species are only stable under high CO-partial pressures and low temperatures and agglomerate to form non-supported Co-particles upon releasing the CO-pressure.^29–31^ In the presence of methanol, Co_2_(CO)_8_ can disproportionate to [Co^II^(HOCH_3_)_6_][Co(CO)_4_]_2_.^30,31^ In the presence of hydrogen and methanol, Co_2_(CO)_8_ is easily hydrogenated to the acidic complex HCo(CO)_4_, this complex is known to catalyse CO-hydrogenation reactions in the liquid phase.^31^ The formation of AADMA and MeOAc is catalysed by the acidic complex HCo(CO)_4_. The acidity of the cobalt-hydrido species also allows the formation of small quantities of dimethyl ether *via* the dehydration of methanol. The investigated heterogeneous catalysts act as a cobalt reservoir, which continuously provides a certain concentration of cobalt in the methanolic solution. The ICP-OES data of the above-mentioned screening experiments, comparing the freshly prepared and spent catalysts, clearly show a loss of cobalt in almost all cases (see Table 3). Considering more precisely the most active system, based on cobalt/gold, the loss of the promotor metal was higher than that for the remaining materials. The observed depletion, already after the first run, suggests weakly bound Au species easily suspended in the first run, leaving behind more strongly bound Au clusters, which remain in the heterogeneous catalyst even after a second run (Au loading staying constant). Unlike the decrease of cobalt loading, which could be detected as dissolved cobalt species in the methanolic solution, the reduction of the gold loading in the material could not be elucidated by applying the ICP method to the liquid phase (see Table S1†). For the PtCo system, a time-resolved measurement of the leached cobalt amount showed an increase in dissolved cobalt starting from 177 μg mL^−1^ after 3 h to over 390 μg mL^−1^ after 45 h (see Table S2†).

**Table 3:** The loss of cobalt and noble metals during the reaction measured with ICP

Catalyst	Freshly prepared catalyst		Spent catalyst		Spent catalyst (after the second round)	
	X-loading (wt%)	Co-loading (wt%)	X-loading (wt%)	Co-loading (wt%)	X-loading (wt%)	Co-loading (wt%)
Co/Al_2_O_3_	—	9.6	—	9.6		
Ru/Co/Al_2_O_3_	0.33	9.4	0.24	8.9		
Pd/Co/Al_2_O_3_	0.89	11.2	0.93	9.8		
Pt/Co/Al_2_O_3_	0.93	8.9	0.98	7.7		
Au/Co/Al_2_O_3_	0.80	9.0	0.60	7.9 (−12%)	0.60	4.9 (−38%)

**Chart 1:**
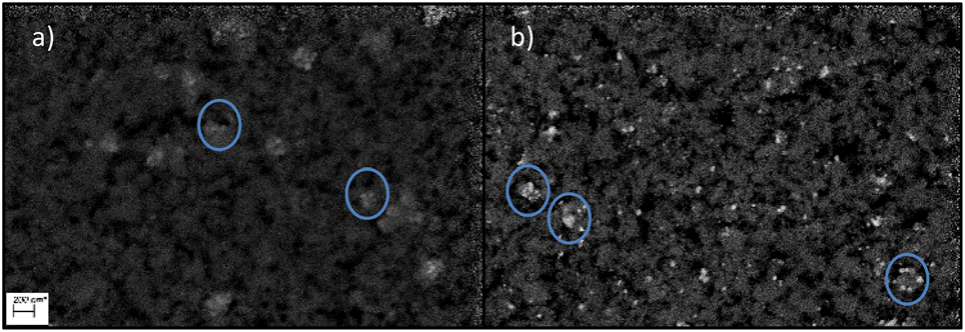
Pre- and post-mortem SEM pictures of AuCo on γ-Al_2_O_3_ at 200 nm scale. Picture (a) shows the freshly prepared catalyst and picture (b) shows the post-mortem catalyst.

In order to access the extent of the cobalt leaching, a freshly prepared catalyst was used in the above-mentioned reaction and then “recycled”. The “recycled” and freshly reduced gold-promoted cobalt catalyst was used a second time under similar conditions and showed a greater loss of cobalt than for the first run (from 12% loss in the first run to 38% loss in the second run, see Table 3). The higher concentration of cobalt in the methanolic solution in the second run caused a higher activity. Pictures of the cobalt particles taken with scanning electron microscopy (SEM) showed a significant agglomeration of the cobalt particles (see Chart 1).

**Fig. 3:**
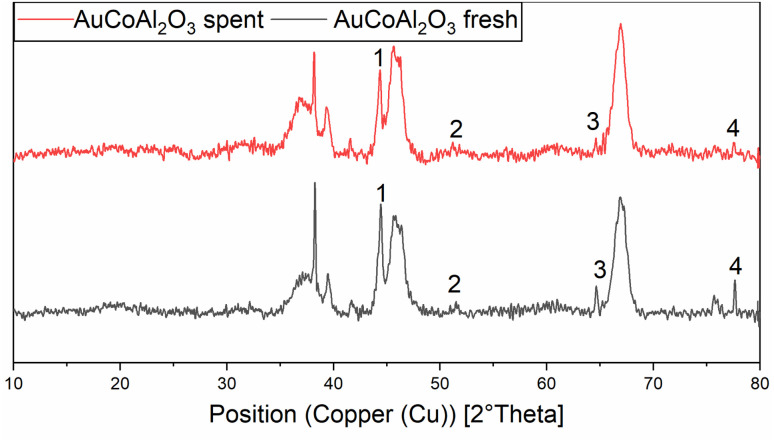
Normalised XRD diffractogram of fresh (black line) and spent (red line) AuCo on γ-Al_2_O_3_ catalyst. 1 = (111) Co-phase, 2 = (200) Co-phase, 3 = (220) Au-phase, 4 = (220) Co-phase.

This again shows the significant mobilisation of cobalt under high CO-partial pressures. Cobalt becomes highly mobile on the surface of alumina and is able to leave the surface as unsupported particles or as soluble carbonyl complexes. The structural change of the catalyst has an incidental impact on the rate of methanisation. Assessing in particular the methane productivity at the end of the reaction clearly indicates higher amounts in the case of the re-used catalyst compared to the freshly prepared one. This dynamic equilibrium, between the dissolved cobalt complexes and cobalt nanoclusters, favours the formation of larger particles due to the minimisation of the surface energy.^32^ The agglomeration and the loss of cobalt loading on the catalyst and of the related crystallite phases can be seen in the XRD diffractogram of the pre-and post-mortem catalyst (see Fig. 3).

**Scheme 4:**
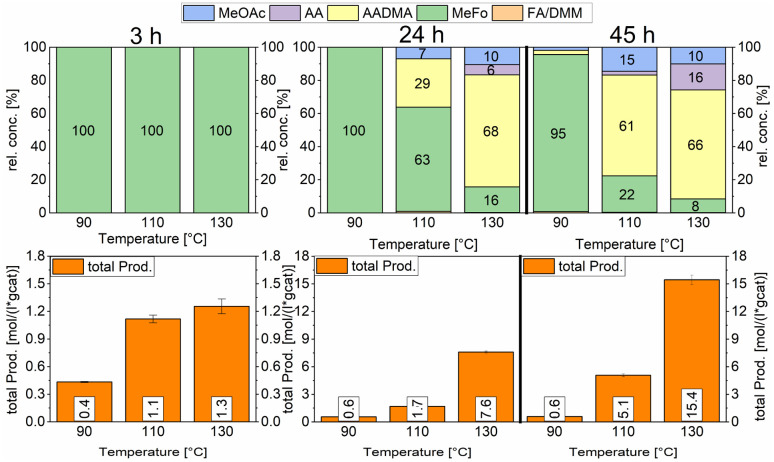
Comparison of total productivity and relative concentrations of the Co_2_(CO)_8_ complex at different temperatures. Testing conditions: 80 bar, CO : H_2_ 1 : 3, 50 mL methanol, quantification: GC-FID (PolyArc), 0.03 g of catalyst.

Both catalysts were compared in the reduced state. The γ-Al_2_O_3_ peaks stayed unchanged, whereas the cobalt peaks for the freshly prepared catalyst were broader which hints at a smaller crystallite size. The fewer and less intense peaks are caused by the loss of cobalt loading and the agglomeration of smaller cobalt particles, which merge different crystallite phases together. For instance, an intensity loss for the peak of the (220) cobalt crystallite phase can be clearly noticed in the diffractogram (Fig. 3). As reported in the literature, cobalt tends to form stable 18 valence electron complexes in the presence of suitable ligands, such as dicobalt octacarbonyl (Co_2_(CO)_8_), as it is likely to be the case under a CO atmosphere.^30,31^ In order to estimate the catalytic activity, Co_2_(CO)_8_ was used as cobalt source and the reaction investigated at different temperatures. Using this complex in the CO-hydrogenation in methanolic solution showed nearly no activity towards the homologation reactions of methanol at 90 °C whereas an increase of the temperature from 110 to 130 °C led to an increase of the catalytic activity (see Scheme 4). The sole product observed at the initial state of the reaction is methylformate (MeFo). Interestingly, this side product was not observed using the heterogeneous catalysts.^30,31^

**Scheme 5:**
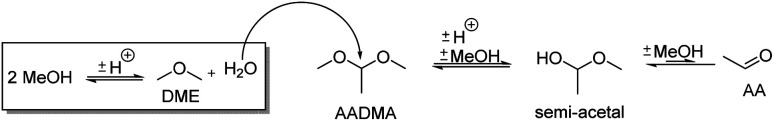
Effect of the methanol condensation reaction on the formation of acetaldehyde (AA) and its semi-acetals.

During the activation of Co_2_(CO)_8,_ the formation of methyl formate in small amounts was observed. After this, the initial state of the MeFo production stops, and only products from the homologation of methanol were observed. The leaching most likely generated HCo(CO)_4_, which is the active species involved in the reactions performed directly with the supported cobalt particles. This is supported by the fact that the heterogeneous catalyst is active in the homologation reaction already at 90 °C (see Scheme 5).

**Scheme 6:**
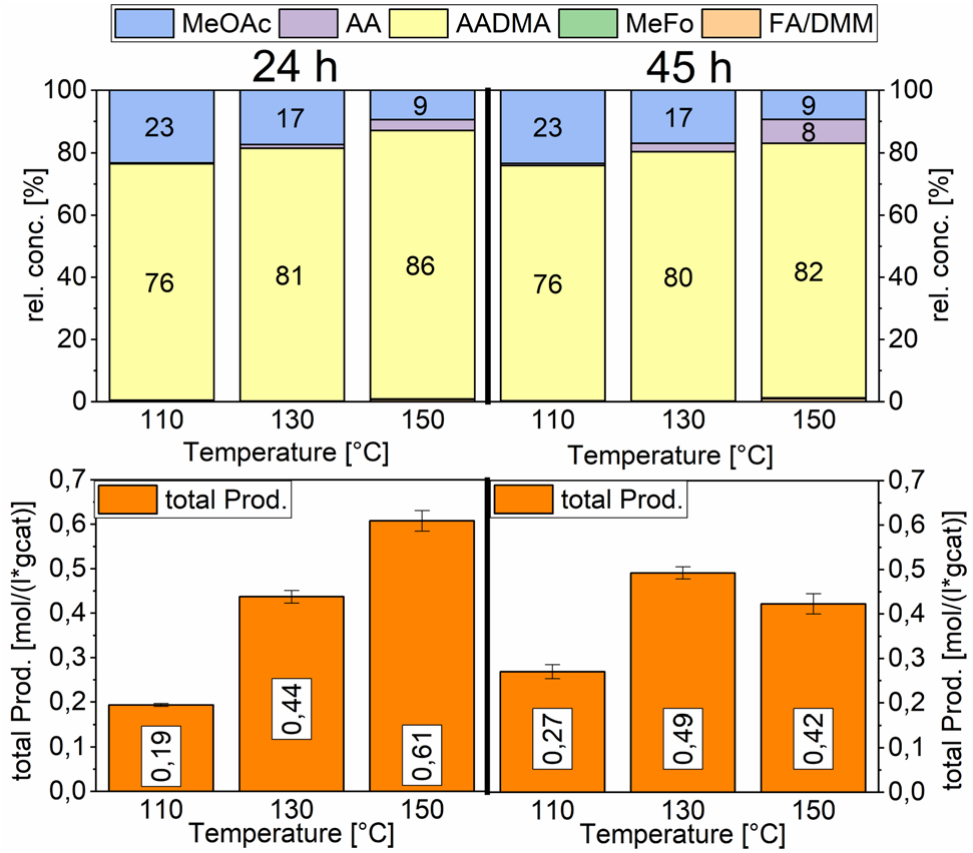
Comparison of total productivity and relative concentrations of the Pt-promoted Co/Al_2_O_3_ catalyst at different temperatures. Testing conditions: 80 bar, CO : H_2_ 1 : 3, 50 mL methanol, quantification: GC-FID (PolyArc), 0.5 g of the catalyst.

**Scheme 7:**
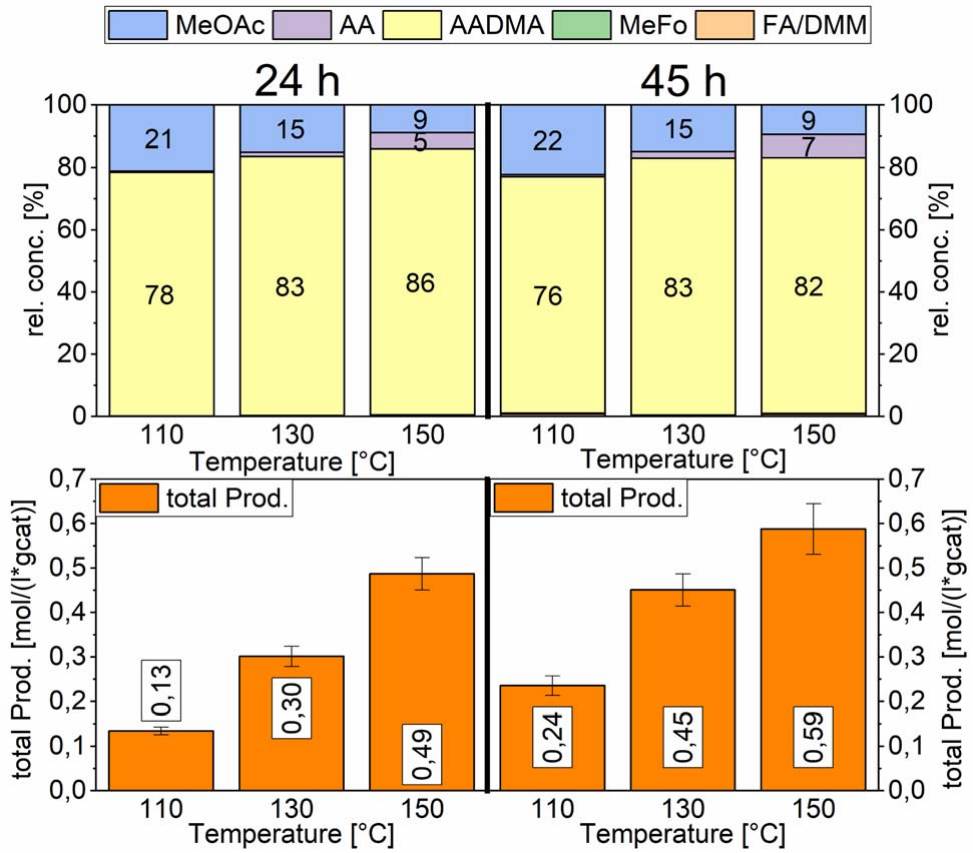
Comparison of total productivity and relative concentrations of the Au-promoted Co/Al_2_O_3_ catalyst at different temperatures. Testing conditions: 80 bar, CO : H_2_ 1 : 3, 50 mL methanol, quantification: GC-FID (PolyArc), 0.5 g of the catalyst.

Temperature variations for the platinum- and gold-promoted catalysts were conducted (see Schemes 6 and 7). They showed a steep increase in activity with the appearance of acetaldehyde as a secondary product from the hydrolysis of AADMA. Dimethyl ether (DME) is also a product generated during this reaction, as described earlier, but it cannot be unequivocally quantified with the setup presented here. At higher temperatures, the DME formation increases, which logically correlates with the increasing water concentration. Water reacts with the acetal bond of AADMA and leads to a hemiacetal of acetaldehyde and methanol. Thermodynamically it is clearly easier for the hydrolysis of acetals to proceed than that of the ethers. All these reactions are part of a reaction network, which produces more hemiacetal and therefore more acetaldehyde when the water concentration in the reaction mixture increases (see Scheme 5).

The overall activity of platinum- and gold-promoted cobalt catalysts supported on alumina is comparable. The reaction temperature has a great effect on the general activity of the catalysts, an increase in temperature causes a steep increase in the reaction rate. At 150 °C, the highest temperature tested, the equilibrium is reached within the reaction time of 45 h (see Scheme 6). Interestingly the Pt-promoted catalyst reaches its equilibrium earlier and the concentrations of AADMA, acetaldehyde (AA), and methyl acetate (MeOAc) start dropping between 24 and 45 h. This is not the case for the Au-promoted catalyst, where side reactions are repressed, but the catalyst reaches its equilibrium concentration later. The CO-conversion reached the equilibrium around 30–40% and the equilibrium methanol conversion around 3.5 to 4.0%. One should notice that the low observed methanol conversion is due to the fact that methanol was used as a reactant without dilution as it has been performed in the literature.^7^

#### Role of the support

**Scheme 8:**
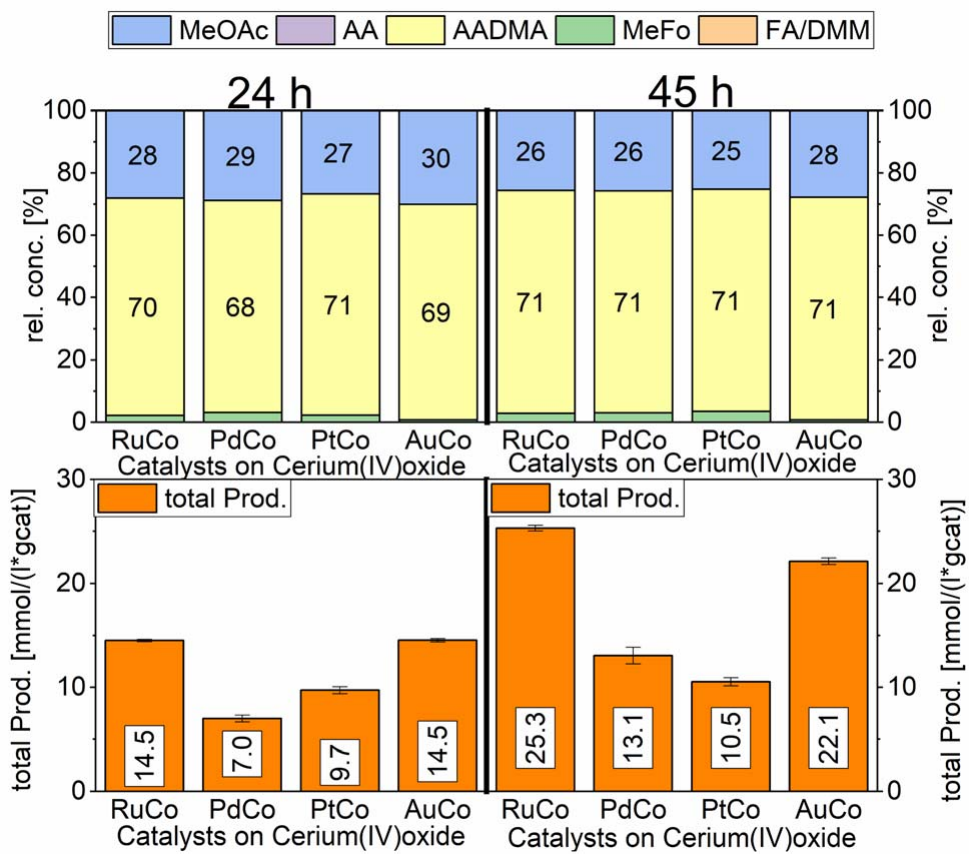
Comparison of total productivity and relative concentrations of Co/CeO_2_ co-impregnated with different promoter metals. Testing conditions: 80 bar, CO : H_2_ 1 : 3, 50 mL methanol, 90 °C, quantification: GC-FID (PolyArc), 1 g of the catalyst.

To increase the metal-support interactions and therefore reduce the amount of cobalt being lost during the catalytic reaction, we varied the support from alumina to cerium(iv)oxide, which has a stronger metal-support interaction due to the redox chemistry of Ce(iv)/Ce(iii).^33^ The selectivities towards MeFo, AADMA, or MeOAc are comparable for all catalysts. The activity tests performed with the cerium(iv) oxide-supported cobalt catalysts showed a general decrease of activity compared to the alumina-supported catalysts, the Ru-promoted one having the highest activity followed by the Au-promoted catalyst. Interestingly palladium promoted cobalt catalyst supported on CeO_2_ had higher activity than the palladium-promoted cobalt catalyst supported on γ-Al_2_O_3_ (PdCo on CeO_2_: 7.0 mmol (L^−1^ g_cat_^−1^), PdCo on Al_2_O_3_: 1.7 mmol (L^−1^ g_cat_^−1^) after 24 h). That can be tentatively explained by the formation of stable cobalt aluminate domains in the case of Pd/Co on an alumina catalyst. These structures need typically high reduction temperatures to form active metallic cobalt regions. Consequently, the concentration of metallic cobalt on the Pd/Co/Al_2_O_3_ catalyst is lower than the concentration of metallic Co on the Pd/Co/CeO_2_ catalyst. The relative concentrations for MeOAc, AADMA, and MeFo are comparable to the alumina-based systems and do not differ by varying the noble metal. This trend suggests again, that the same active species play a role in the catalysis, being just generally less concentrated in the solution, because of stronger metal-support interactions in the case of ceria-supported catalysts (Scheme 8).

#### Pressure variation

**Scheme 9:**
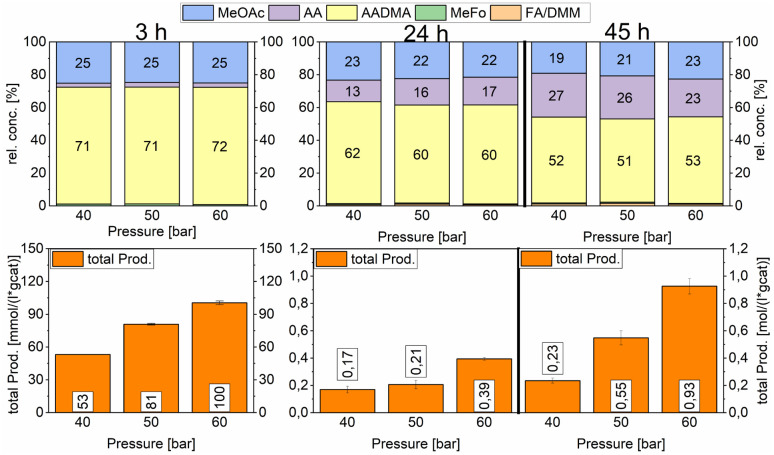
Pressure variation for the AuCo on γ-Al_2_O_3_ catalyst. Testing conditions: 150 °C, CO : H_2_ 1 : 1, 50 mL methanol, quantification: GC-FID (PolyArc), 0.5 g of the catalyst.

Modifying the starting pressure had a direct effect on the general activity already after 3 h. After filling the reactors with the catalysts and methanol under inert conditions, the reactors were first pressurised up to 40 bars with a mixture of CO and H_2_ in a 1 : 1 ratio (vol%). The second reactor was further pressurised with argon to 50 bars and the third reactor to 60 bars. After heating the mixture to reach 150 °C, the reaction was monitored and liquid samples were taken over several hours to compare the activities of a specific catalyst. The relative concentrations of the products were similar for all tested pressures. Acetaldehyde concentration increased with the reaction time and reached its maximum after 47 h with around 25% of the relative concentration. As described earlier this happens due to the fact that the equilibrium conversion of the methanol condensation reaction is reached and all consecutive reactions are therefore repressed (*e.g.* the hydrolysis of AADMA). The highest total productivity of 0.93 mol (L^−1^ g_cat_^−1^) was reached in 47 h only for the 60 bar experiment (Scheme 9).

#### Variation of synthesis gas mixture

**Scheme 10:**
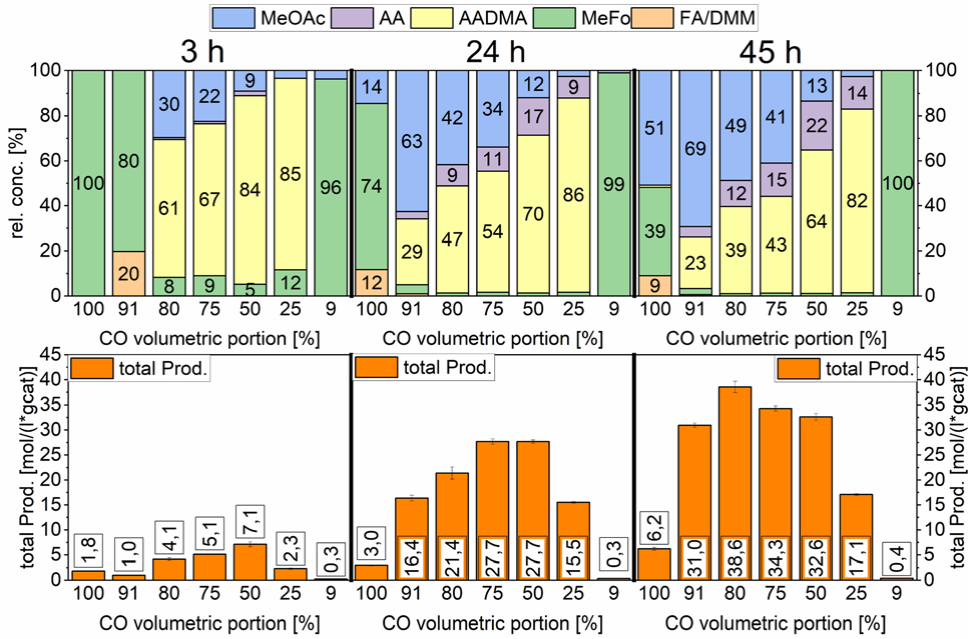
Variation of the volumetric portion of CO in undiluted synthesis gas. Testing conditions: 150 °C, 80 bar, 30 mg of Co_2_(CO)_8_, 1000 RPM, and 50 mL methanol.

To further understand the mechanistic details of the ongoing processes, studies were performed by varying the synthesis gas. The percentages of CO and H_2_ were varied from 100% CO to 9% CO (see Scheme 10). For all the mixtures the reaction was run for 45 h and samples were taken in regular intervals to obtain information about total productivity and relative concentrations of the products. The catalyst used for this study was Co_2_(CO)_8_. At low CO partial pressures (<50%) the carbonyl species' in the methanolic solution were unstable and a black residue of cobalt nanoparticles that accumulated on the reactor walls and stirrer where observed. This is correlated to low partial pressures of CO and accordingly lower amounts of CO dissolved in methanol. This leads to the instability of the cobalt carbonyl species and the unwanted agglomeration of cobalt nanoparticles. Looking at the total productivity of the reactions carried out with an H_2_-rich synthesis gas, the observed low productivity compared to the reactions carried out with a CO-rich synthesis gas indicated that these cobalt particles did not participate in the catalytic reaction at these temperatures.

Furthermore, the CO partial pressure had a great impact on the relative concentrations of all products. At low total productivity, MeFo was always a major product but did not play any role in reactions with higher total productivity. At 80% volumetric portion of CO, the total productivity was the highest (see Scheme 10). The relative concentration of AADMA/MeOAc was directly linked with the CO volumetric portion. When pure CO was used, no AADMA was formed, increasing the H_2_ partial pressure from this point started the homologation reaction of methanol to yield acetaldehyde, also increasing correlatively the relative concentration of AADMA to reach its maximum at 82% using a synthesis gas mixture of 1 : 3 CO : H_2_. Only low amounts of MeFo (1%), MeOAc (3%), and acetaldehyde (14%) were found as side products after 45 h (total prod. 17.1 mol (L^−1^ g_cat_^−1^)). Regarding the relative concentration of MeOAc and MeFo, which are products of the reaction of methanol and CO without H_2_ being involved, the relative concentrations behaved the other way around. At a CO volumetric portion of 91% the highest MeOAc selectivity could be reached at 69% with a remarkable total productivity of 31 mol (L^−1^ g_cat_^−1^) after 45 h. Further increasing the H_2_ partial pressure led to an 80 : 20 of the CO : H_2_ mixture, which showed the highest productivity but also the least selective reaction (38.6 mol (L^−1^ g_cat_^−1^)).

**Scheme 11:**
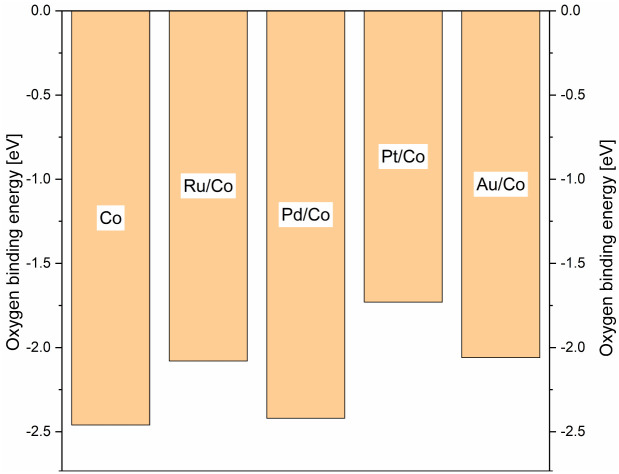
Oxygen binding energies for promoted und non promoted cobalt systems.

**Scheme 12:**
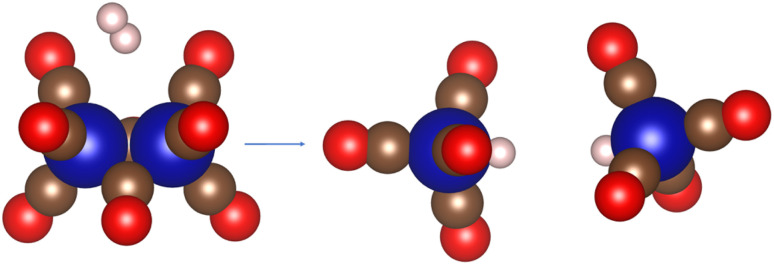
Schematic representation of Co_2_(CO)_8_ hydrogenation process.

Density functional theory (DFT), DFT calculations were performed in order to complement the experimental work and give some information regarding the mechanisms at work, in the solution, and on the surface. In order to find a simple descriptor able to evaluate both promoted and non-promoted cobalt catalysts and correlate with the reducibility of the oxidic materials, we calculated the oxygen binding energy on the 2 × 2 large Co(111) unit cell and its 0.25 mL surface alloy with Pd, Pt, Au, and Ru (see Scheme 11). The oxygen binding energy help understand the calcination step where Co_3_O_4_ species interact with the noble metal promotors leading eventually to materials of different catalytic activities. The highest binding energy was found for the PdCo system, close in value to the pure cobalt system. On the other hand, in the presence of Pt and Au, oxygen binding energy was significantly reduced. As a consequence, the reduction of the PdO/Co_3_O_4_ catalyst was expected to be incomplete, leading to the lowest amount of metallic cobalt at the surface of the catalyst. In contrast, platinum and gold showed a similar and low oxygen binding energy, strongly suggesting that most of the Co_3_O_4_ promoted with Pt or Au will be reduced to metallic cobalt and hence will lead to more active catalysts. The variation of the promotor metal has a direct influence on the reducibility of the cobalt and, under the CO atmosphere, on the mobilisation of the metal and leaching phenomena. Therefore, it seems reasonable to link the difference in the catalytic activity to a varying leaching intensity and not to a direct change in the electronic structure of the heterogeneous catalysts upon co-impregnating the promotor metals. DFT calculations were performed to support assumptions regarding the mechanisms at work, in the solution, and on the surface. Comparing the activity of homogeneous and heterogeneous catalysts at 90 °C, it clearly showed that the heterogeneous catalyst was already active at mild temperature, whereas the Co_2_(CO)_8_ complex barely showed any activity even after 45 h of the reaction time (Scheme 12).

The reaction enthalpy for the reaction of the Co_2_(CO)_8_ cluster and hydrogen to form two HCo(CO)_4_ monomers was calculated to be 26.1 kJ mol^−1^, in very good agreement with the experimental value of 13.4 kJ mol^−1^ to 27.6 kJ mol^−1^ (for details see ESI†).

Cobalt tetracarbonyl hydride has been proposed as the active species in many homogenous cobalt catalysed CO-hydrogenation reactions, for example for hydroformylation.^34^ The initiation step that is usually assumed in the literature for hydroformylation reactions is the removal of one carbonyl ligand and the formation of HCo(CO)_3_, followed by a subsequent alkene and hydrogen adsorption and activation. While the first step of CO detachment is usually uphill in free energy, consecutive steps are typically downhill in energy. We have considered the same initial step for the methanol activation and the formation of CH_3_Co(CO)_3_. As shown in Fig. S5 (see ESI†), this process is uphill in energy for both, the detachment of a carbonyl ligand as well for the consecutive methanol dissociation.

As a second possibility, we have considered a proton transfer from HCo(CO)_4_ to methanol, followed by dissociation into CH_3_Co(CO)_4_ and H_2_O. The reaction-free energy barrier of this process is 135 kJ mol^−1^ (see Fig. S8†). Since the reaction is taking place in the liquid phase, the process could in principle be stabilized by the solvent, in our case, methanol. A rough estimation of this effect was obtained using three methanol molecules in the vicinity of the cobalt complex where the barrier was reduced to 100 kJ mol^−1^. We note, however, that only an in-depth molecular dynamics study would be able to reveal the influence in more detail.

An alternative path for the formation of the H_3_C-[Co] species has also been discussed in the literature.^35^ In the presence of methanol the neutral Co_2_(CO)_8_ dimeric molecule can be disproportionate to cationic [Co(CH_3_OH)_6_]^2+^ and two anionic complexes of [Co(CO)_4_]^−^.^30,31^ We considered a nucleophilic attack of one cobaltate [Co(CO)_4_]^−^ on a methyl group of the cationic complex [Co(CH_3_OH)_6_]^2+^. It is known that the tetracarbonylcobaltate anion is quite nucleophilic.^35^ After removing the hydroxide complex [Co(CH_3_OH)_5_(OH)]^+^ and concomitant formation of a reactive CH_3_Co(CO)_4_, the reaction can proceed further. Furthermore, it is also known that the [HCo(CO)_4_] hydride is a strong acid and will therefore quench the hydroxide complex in solution.^35^ The free energy barrier of this process was calculated to be just 88 kJ mol^−1^ (see Fig. S9†). We, therefore, consider this as the most likely scenario of CH_3_Co(CO)_4_ formation when using Co_2_(CO)_8_.

**Fig. 4:**
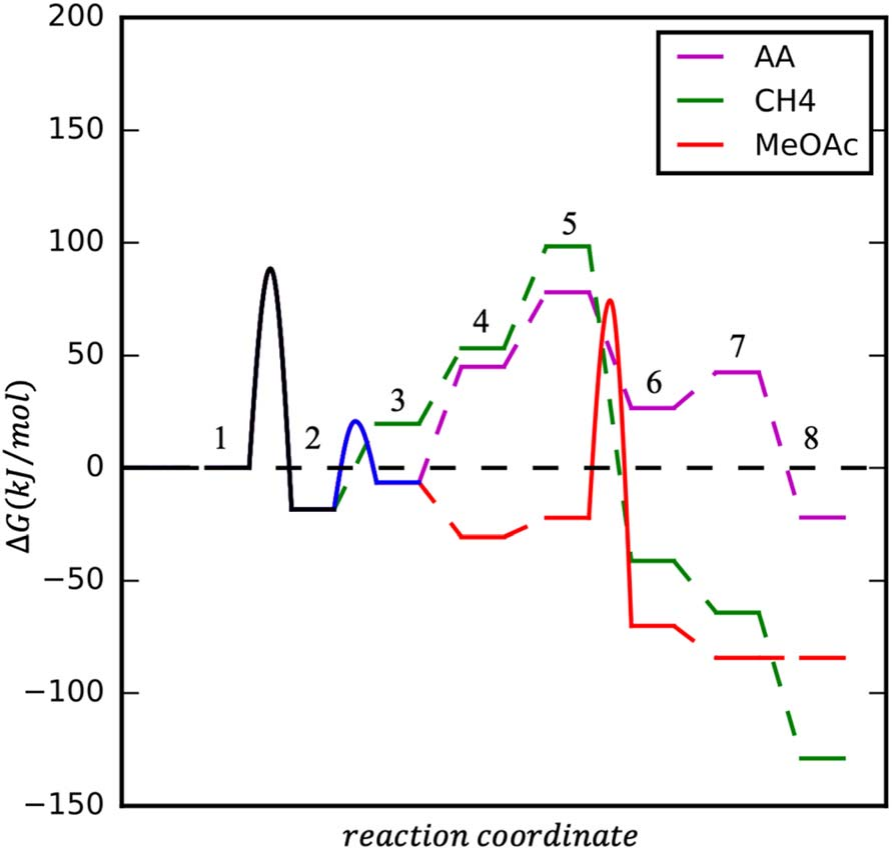
Reaction energy diagram for AA, CH4 and MeOAc formation on HCo(CO)4 at *T* = 423.15 K, p (CO) = 20 bar, p (H_2_) = 60 bar. Starting conditions: (1) 2 x (Co(CO)_4_)[Co(CH_3_OH)_6_] and HCo(CO)_4_, (2) Co(CO)_4_CoOH(CH_3_OH)_5_ + CH_3_Co(CO)_4_ and CH_3_Co(CO)_4_; **(AA)**: (3) CH_3_COCo(CO)_3_, (4) H_2_ + CH_3_COCo(CO)_3_, (5) 2H + CH_3_COCo(CO)_3_, (6) HCo(CO)_3_ + CH_3_CHO, (7) HCo(CO)_3_ + CH_3_CHO (l), 8) HCo(CO)4 + CH3CHO (l); **(CH_4_)**: (3) CH_3_Co(CO)_3_, (4) H_2_ + CH_3_Co(CO)_3_, (5) 2H + CH_3_Co(CO)_3_, (6) HCo(CO)_3_ + CH_4_, (7) HCo(CO)_3_ + CH_4_ (g), (8) HCo(CO)_4_ + CH_4_ (g); **(MeOAc)**: (3) CH_3_COCo(CO)_3_, (4) CH_3_COCo(CO)_4_, (5) CH_3_COCo(CO)_4_ + MeOH, (6) HCo(CO)_4_ + MeOAc, (7) HCo(CO)_4_ + MeOAc (l).

**Fig. 5:**
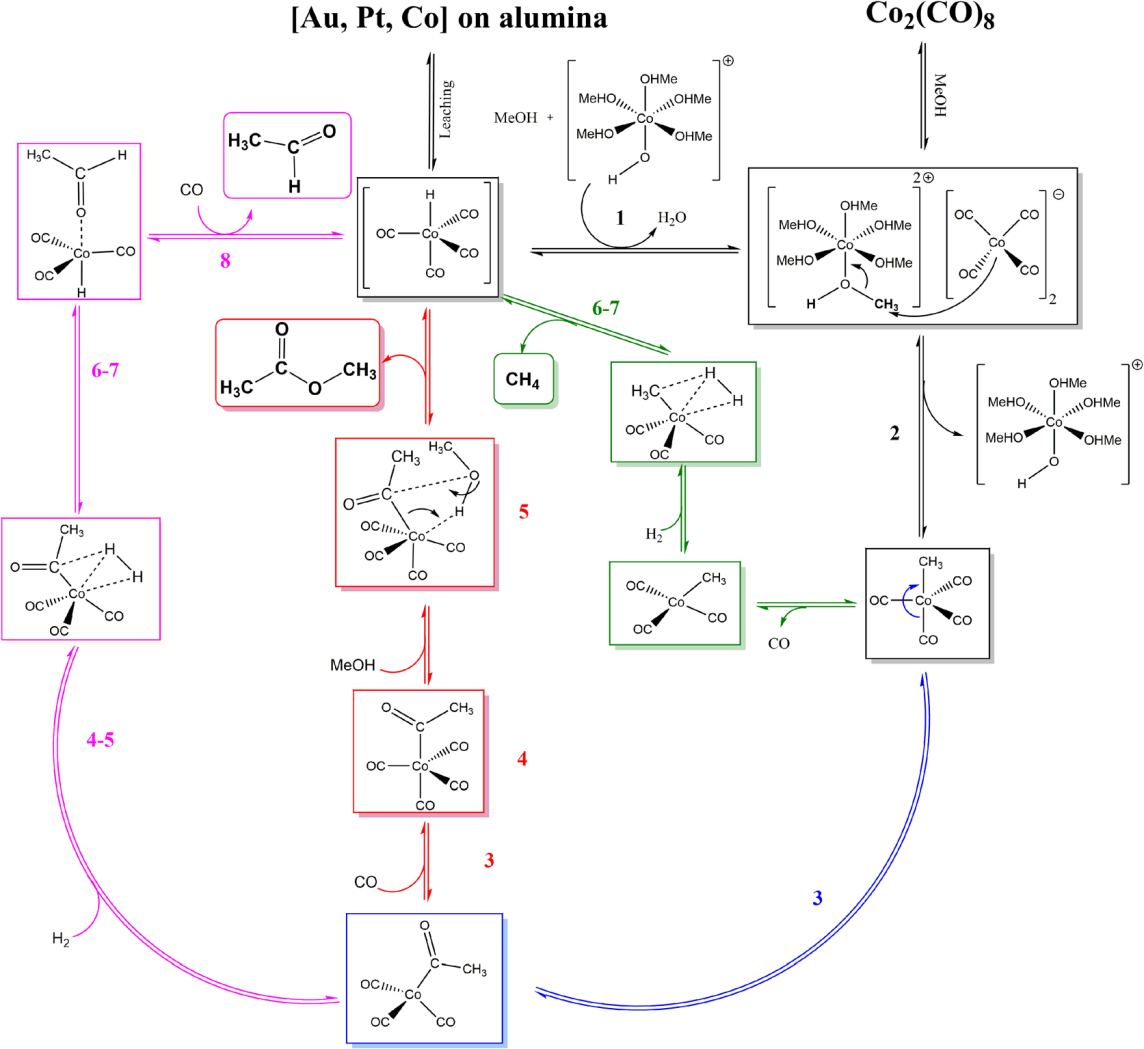
Catalytic cycle according to DFT calculations towards both major products: AADMA and MeOAc. The pathway to CH_4_ is also shown.

Upon CH_3_Co(CO)_4_ formation three processes could take place: CO insertion into CH_3_ and CH_3_CO formation, followed by (1) hydrogen adsorption, activation, and AA formation, (2) methanol activation and MeOAc formation. Instead of CO insertion, the CO removal followed by hydrogen adsorption and activation leads to the formation of CH_4_. Reaction diagrams of the processes are presented in Fig. 4 and the concluding catalytic cycle is shown in Fig. 5.

As mentioned, the formation of CH_3_Co(CO)_4_ could be followed by CO insertion with a small barrier (40 kJ mol^−1^) into CH_3_ and the formation of Co(CO)_3_–COCH_3_. Hydrogen binding is 52 kJ mol^−1^ uphill in energy and is followed by its dissociation. Even though H_2_ dissociation has a low barrier, the final state is not stable due to 6 ligands present at the cobalt center and the almost spontaneous process of hydrogen insertion into acetyl–cobalt bond and acetaldehyde formation takes place (see magenta path Fig. 4 and 5). The presence of methanol in the solution, favoring the formation of the stable acetal, could additionally improve the process.

After the formation of (CO)_3_Co–COCH_3_, the formation of (CO)_4_Co–COCH_3_ occurs by attaching an additional CO ligand. Methanol can interact with the acetyl group in a concerted mechanism (see red path Fig. 4 and 5).

Upon the formation of CH_3_Co(CO)_4_, the removal of one CO ligand could take place, this process being only 38 kJ mol^−1^ endothermic. Hydrogen adsorption is slightly endothermic with 34 kJ mol^−1^ but its activation requires a slightly higher barrier than on Co(CO)_3_–CH_3_CO. The overall process for methane formation is slightly less favourable than for acetaldehyde and methyl acetate formation. Considering that CH_4_ is a gas and the other products are liquids at the reaction conditions and are also further stabilised in the protic solvent, the formation of MeOAc and AA is favoured over the formation of CH_4_ (see green path Fig. 4 and 5).

In summary, the calculations suggest that among the three processes, AA and MeOAc formation, although they have similarly high barriers, AA formation has a smaller overall difference between the lowest and highest energy levels, giving it an advantage for the formation. The methane formation has a higher barrier and is expected to be formed in smaller amounts.

## Experimental

### Materials

Chemicals and materials were supplied by: Alfa Aesar^A^, abcr GmbH^abc^, Merck KGaA^M^, Strem Chemicals^S^, Sigma-Aldrich^SA^, Air Liquide^AL^ and Thermo Scientific^TS^ and used without further purification. The values in brackets show purity and other properties.

Hexachloroplatinic acid hexahydrate (99.9%)^A^, ruthenium(iii)chloride hydrate (99.9%)^abc^, tetrachloroauric acid hydrate (99.9%)^A^, palladium(ii)nitrate hydrate (99.8%)^A^, cobalt(ii)nitrate hexahydrate (97.7%)^A^, nitric acid (65%)^M^, gamma-aluminium oxide (99.97%, 80–120 m^2^ g^−1^)^A^, cerium oxide (99.995%, nanopowder <25 nm)^SA^, dicobaltoctacarbonyl (stabilised with 1–5% hexane)^S^, carbon monoxide (99.97%, 200 bar, 40 l)^AL^, hydrogen (99.999%, 300 bar, 50 l)^AL^, argon (99.9999%, 200 bar, 50 l)^AL^, methanol (99.8, extra dry over molecular sieves)^TS^.

### Synthesis of heterogeneous catalysts

Metal precursors (H_2_PtCl_6_·6H_2_O, RuCl_3_·*x*H_2_O, HAuCl_4_ H_2_O) were dissolved in water (10 wt% HNO_3_ for Pd(NO_3_)_2_·H_2_O) and the precalcined support was added. Afterwards, the cobalt precursor (Co(NO_3_)_2_·6H_2_O) was added in small portions. The suspension was stirred overnight at 60 °C, the water was removed using a rotary evaporator and the so obtained catalysts “chunks” were dried in a static oven at 120 °C. After cooling to RT and grinding, the so received powders were calcined at 600 °C for 6 hours at a heating rate of 10 K min^−1^. The calcined powders were reduced *ex situ* before being used in the catalyst testing apparatus. The reduction was run for 5 h at 400 °C, using a 5 vol% H_2_ in N_2_ mixture (4 L h^−1^) and a heating rate of 7 K min^−1^. The temperature was kept at 400 °C for an additional hour, the catalysts were then continuously purged with argon and allowed to cool down to room temperature. This procedure is based on the literature.^36,37^

### Powder X-ray diffraction (PXRD)

PXRDs were recorded on a PANalytical X'Pert Pro X-ray diffractometer (Bragg–Brentano geometry with Cu Kα radiation and a Ni filter). The range between 5° and 80° was measured within 2 h. The diffraction patterns were compared to reference compounds from the Joint Committee of Powder Diffraction Standards (JCPDS) database. The samples were measured both in calcined and reduced states. Minimal exposure to air during the measurements of the reduced catalysts could not be avoided due to the used setup, but the trends for the crystallite size determination were still meaningful. The observed peaks of the calcined and reduced catalysts were assigned based on JCPDS ref. 38–45.

The crystallite size of Co_3_O_4_ was estimated by using the Scherrer equation,^46^ for the most intense Co_3_O_4_ peak (*i.e.* at 2*θ* = 36.9°). The Co_3_O_4_ crystallite size was obtained by assuming spherical particles and correcting the crystallite size obtained from the Scherrer formula.^47^ The K-α 1 line and a K-factor of 0.90004 was used for calculations. The particle size for metallic cobalt was then calculated with eqn (1). Cobalt oxide particles may undergo structural modifications (cracking) during the reduction in H_2_ and in such a case eqn (1) is not directly applicable.^23^

1 *d*(Co^0^) = 0.75 × *d*(Co_3_O_4_)

The conversion of Co_3_O_4_ to Co particle size was performed according to the relative molar volumes.^22^ The peaks were fitted with X'Pert HighScore and the line broadening of the instrument was calculated from a calibration sample containing lanthanum hexaboride.

### Inductively coupled plasma atomic/optical emission spectroscopy (ICP-OES)

The calcined samples (50 mg) were analysed for their Co, Pt, Au, Pd, and Ru contents. The samples were treated in a dedicated microwave oven with reverse aqua regia (3 : 1 HNO_3_ to HCl) to dissolve the samples completely. The so obtained solutions were directly used for analysis on Agilent 700 series ICP optical emission spectrometers.

### X-ray fluorescence spectroscopy

In contrast to ICP-OES, XRF can directly analyze a wide range of elements without time-consuming preparation techniques. The calcined samples (200–500 mg) were analysed for their Co, Al, Pt, Au, Pd, and Ru contents. The samples were measured on a Bruker S4 Pioneer spectrometer, preliminarily prepared as powders on a Mylar foil, and using a dedicated 34 mm collimator mask for the measurements.

### Catalytic activity tests

Further comparison of the catalysts was performed with a high-pressure parallel screening apparatus. The so-called “PASCAR” (for PArallel Screening of CAtalytic Reactions) plant is a 3-folded batch reactor plant that can be used to evaluate catalytic reactions and monitor the formation of specific compounds *via* off-line analytics, the reactions taking place ideally in the liquid phase. The three reactors have separate gas (H_2_, CO, and Ar) and liquid dosing systems. Details about the used procedures and setup can be found elsewhere.^36^

### Process analytics

For the analysis of the products, a specific offline-GC FID (Agilent Technologies GC 8890) with an autosampler (50 Position Autoinjector, Agilent Technologies G4567A) was used. The use of a dedicated Dean's switch device is mandatory in order to protect the detector from a methanol overload (as we are systematically working with methanolic solutions). The injection temperature was kept at 180 °C, with a split ratio of 50 : 1. The starting temperature for the oven was 40 °C, which was held for 2 min and then heated at 25 K min^−1^ to 180 °C, the columns used were a combination of DB-wax ultra inert (30 m, 0.32 mm, 0.5 μm, Agilent Technologies) and a deactivated column after the Dean's switch. A total run time of 7.6 min allowed a fast analysis of liquid samples and gave good separation for methanol, dimethoxymethan, methylformate, formaldehyde, acetaldehyde, acetaldehyde dimethyl acetal and methylacetate. In addition, traces of propionaldehyde dimethyl acetal and propionaldehyde diethyl acetal were observed but not quantified. Fischer–Tropsch products could not be measured. Details about process analytics have been published elsewhere.^36^

### Density functional theory calculations (DFT)

Density functional theory (DFT) calculations were performed using the Vienna *ab initio* simulation package (VASP)^48,49^ in connection with the atomic simulation environment (ASE).^50,51^ The bayesian error estimation functional with van der Waals correlations (BEEF-vdW)^52^ with the projector augmented wave method (PAW)^53^ and a plane-wave basis set with a cutoff energy of 450 eV were used. Due to the presence of delocalized Co d orbitals, we applied GGA + *U* (*U* = 4.0 eV) method (more details in ESI†).^54^

Large 12 × 12 × 12 Å unit cells were used to represent isolated HCo(CO)_4_ species and 30 × 15 × 15 Å unit cells were used to represent 2 × Co(CO)_4_ [Co(CH_3_OH)_6_] species. The Brillouin zone was sampled using a 3 × 3 × 3 Monkhorst–Pack *k*-point grid for smaller and 1 × 2 × 2 for larger unit cells. The convergence criterion for the geometry optimisations was a maximum force of 0.01 eV Å^−1^. Transition states are obtained using constrained optimisations and Nudge elastic band (NEB)^55^ calculations. All transition states were verified to contain a single imaginary harmonic frequency corresponding to the transition vector of the reaction. Entropic contributions to the free energy were calculated within the harmonic approximation for adsorbates, and entropic contributions for gas-phase species were obtained from tabulated values (see ESI† for all data). All optimised structures of adsorbates and transition states are given in the ESI.†

## Conclusions

The heterogeneously catalysed homologation of methanol to C_2_-compounds such as acetaldehyde and its acetals although initially promising, was revealed to be a tedious process in practical terms. Using high pressures of the synthesis gas led to a high mobility of the active carbonyl species in the reaction medium. Classical immobilisation techniques lead to. *e.g.* stronger metal–support interactions resulting in an activity loss, which incidentally suggested that the process at work in this reaction is mostly homogenously catalysed. The heterogeneous catalysts used in this study, involving metallic cobalt supported on alumina, acted mostly as a cobalt “reservoir”, leaching active cobalt species into the methanolic solution, where the homologation reaction actually takes place in a homogeneous way. The relatively low temperature, high partial pressures of CO, and the presence of a protic liquid phase greatly enhanced the formation of such cobalt carbonyl species. Such an approach has been used with success using epoxy resins as the matrix.^56^ The reaction conditions vastly change the structure of the catalyst as could be seen during the characterisation of the spent catalysts. A strong agglomeration of metal particles and a loss of the cobalt loading were noticed for all catalysts investigated in this study. A strong dependency on the catalytic activity and the nature of the promotor metal could be observed. Even at low loadings of 1-to-10 related to cobalt, the promotor has a great effect on the reducibility, the structure, and consequently on the activity of the catalyst. This study showed that an increase in temperature did not lead to unwanted side reactions at 150 °C, with the gold-promoted cobalt catalyst supported on alumina showing remarkable activity and selectivity towards AADMA. First, facile investigations of the ongoing processes could shed light on the difference in the catalytic activity of Co_2_(CO)_8_ and supported cobalt catalysts, but further more detailed investigations are necessary to understand the leaching process in detail. A vast variation of CO partial pressure in the CO/H_2_ mixture gave interesting insights into the reaction mechanism, regarding the effect of hydrogen partial pressure on the relative concentrations of the products and the total productivity. Reaction barriers and different pathways to key intermediate species were considered with the help of DFT calculations. These calculations revealed different possible pathways towards the major products and key intermediates consisting of the disproportionation step, followed by the formation of the methyl–cobalt bond, which is the key intermediate. From this intermediate the formation of all three products was calculated in detail, starting from the CO–insertion into the methyl–cobalt bond as a first step towards AA and MeOAc formation or CO detachment as a step to the formation of CH_4_.

## Author contributions

K. A. Sheikh: catalyst synthesis, characterisation, testing, calibration, maintenance of process analytics, visualisation, original draft, review, and editing. T. A. Zevaco: visualization, evaluation of characterisation, supervision, writing, reviewing, and editing. J. Jelic: calculations and modelling, density functional theory calculations, visualisation of results, writing, reviewing, and editing. F. Studt: conceptualisation, supervision, investigation, writing, reviewing and editing. M. Bender: funding acquisition, conceptualisation, supervision, investigation, writing, reviewing, and editing.

## Conflicts of interest

There are no conflicts to declare.

## Supplementary Material

RA-013-D3RA02784H-s001

RA-013-D3RA02784H-s002
