# Supplementary material for: Efficient noble metal promoted bimetallic cobalt catalysts in the selective synthesis of acetaldehyde dimethyl acetal
Source: RSC Adv. 2023 Jul 26;13(33):22698–709. doi: 10.1039/d3ra02784h (PMC10369129; doi:10.1039/d3ra02784h)
Supplement: RA-013-D3RA02784H-s002 [file RA-013-D3RA02784H-s002.pdf]

Electronic Supplementary Material (ESI) for RSC Advances.
This journal is © The Royal Society of Chemistry 2023

Supplementary Information (SI)

Efficient noble metal promoted bimetallic Cobalt Catalysts in the selective Synthesis of Acetaldehydedimethylacetale

K. A. Sheikh[a],[b], T. A. Zevaco[a], J. Jelic[a], F. Studt[a], M. Bender[b]
[a]Karlsruher Institut für Technologie (KIT), [b]BASF SE

Standard procedure for catalyst screening:

1 g of catalyst was weighed into the autoclave under inert gas flow. The autoclave was closed and filled with argon to 2 bar and released, this process was repeated three times. After that the HiBatch receipt was started. Generally, the receipt starts after the user admits to have performed all precautions before starting the experiment. Like opening the pressure equalisation valve for the methanol container, opening manual gas inlet valves for CO, H_2 and Ar, and opening the gas cylinders. After that the counters for the amount dosed of CO and H_2 are resetted for all three reactors. Followed by the adjustment of the multivalve into the Position 1 (for reactor 1). The Multiportvalve controls the flow of the solvent to each of the reactor. After adjusting it, the liquid dosing starts (User has to give amount of methanol needed). Simultaneously the gas dosing starts, where the user has to give the maximum pressure value, stirrer speed (and ramp) and the needed mixture of H_2 and CO. After that the Mass-Flow Controllers (MFC) for H_2 and CO are started. All setpoints for the MFCs are reached with a ramp. This prevents overshooting of the MFC. Still we dose the mixture into the fume hood until the MFCs are in a steady state. After that the user has to give the permission and the valves towards the fume hood close and the valve towards the reactor open and the gas dosing starts. The gas dosing can be controlled with the total amount of CO/H_2 dosed (in ml) or with the total pressure needed (in bar). All runs in this study where performed with the total pressure variant. After the end of liquid dosing the receipt automatically switches to the next reactor. After the end of gas dosing the system automatically stops the dosing, switches of the MFCs, closes the valves and starts the heating procedure (Reactor inlet temperature regulation and Jacket temperature regulation is possible).

PASCAR plant:

The plant is controlled with 21 pneumatic valves. This allows a separate dosing of CO and H_2 into each reactor. Additionally one Argon MFC is used for the purging of the reactors with intert gas. It can also be used as a pressure control. As the use of reactant gas reduces the reaction pressure, fresh synthesis gas (CO/H_2) or inert gas can be dosed into the system to keep the pressure of all reactors constant all throughout the experiments. Most experiments are run for 24 h or 45 h. A mechanical stirrer can stir the mixture up to 1200 RPM. Inlet pressures of the gases and the reactor pressures are monitored. The inlet temperature and the mantle temperature is recorded separately. Both temperatures can be regulated. Furthermore hexane, dimethoxymethane, methanol and other solvents can easily exchanged and dosed with two available HPLC. Therefore also mixtures of these solvents can be dosed. The catalyst have to be introduced before closing the reactors. Liquid catalysts or catalyst solutions can also be dosed with the HPLC pump. Beside liquids, gaseous compounds can be dosed in total amounts. Therefore, one gaseous compound can be dosed and the mixture heated to the desired temperature, after reaching the temperature the second (reactive) gaseous compound is dosed into the reactor, which marks the starting point of the reaction. For slower reactions starting points can be marked by the time the reaction temperature is reached.

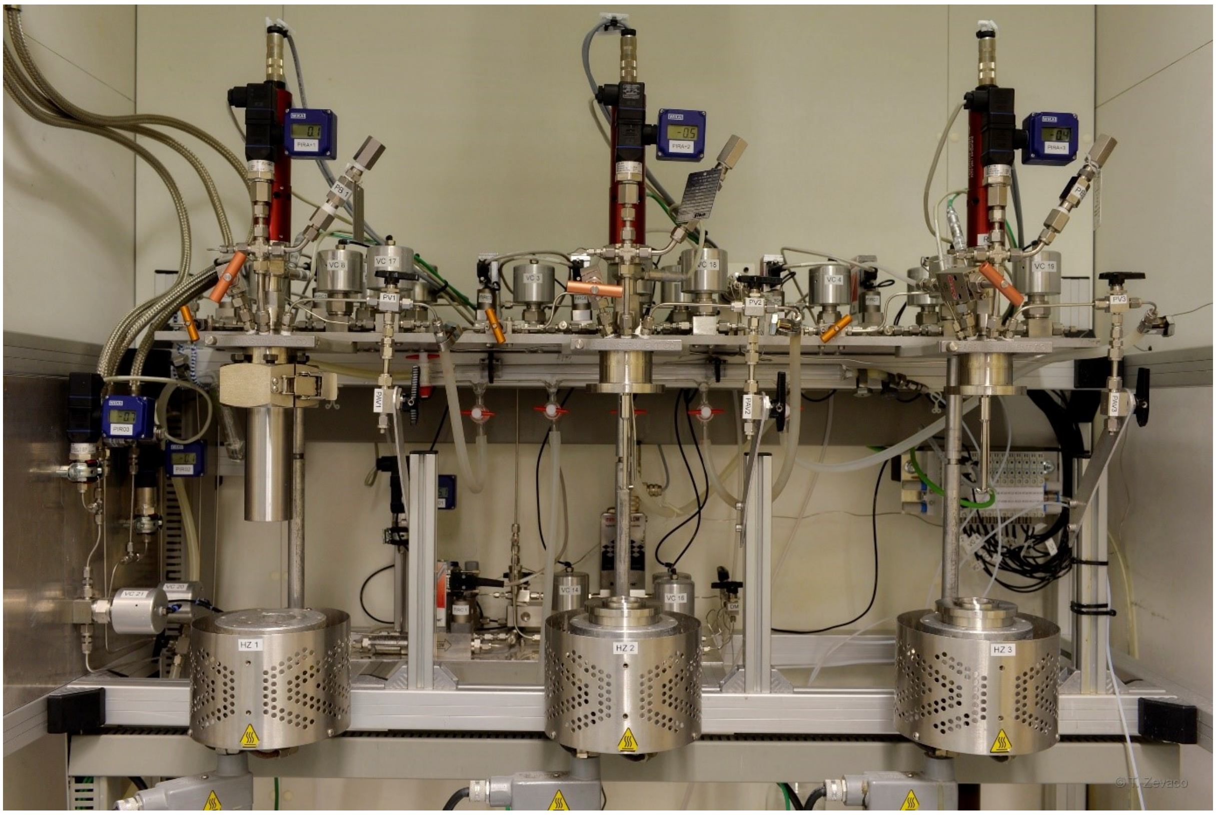

Figure S1: Picture of the PASCAR plant showing all three reactors.

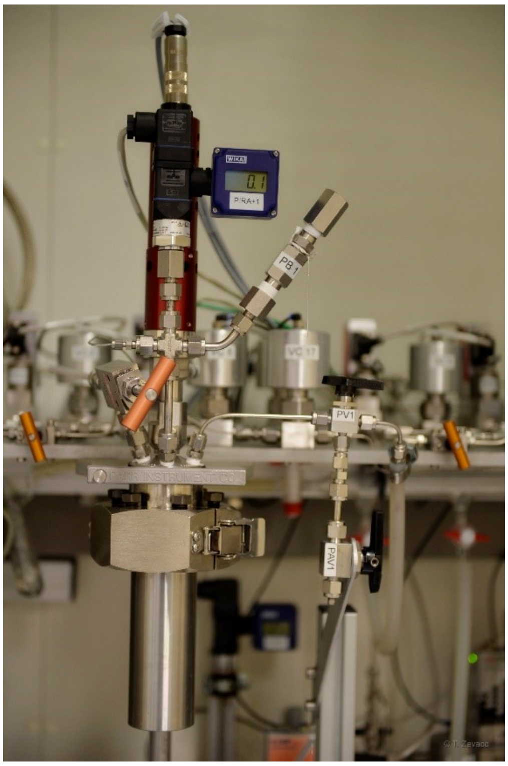

Figure S2: Close up picture of one reactor of the PASCAR plant and the sample loop.

ICP-OES Results of the recycling runs

Table S1: Results of the ICP measurements of the methanolic solutions after the reaction. The values in the brackets show the cobalt concentration on the second run.

Sample	Ru_ICP µg/ml	Co_ICP µg/ml	Pd_ICP µg/ml	Pt_ICP µg/ml	Au_ICP µg/ml
Co on Al_2O_3	-	119.4	-	-	-
RuCo on Al_2O_3	8	137.5	-	-	-
PdCo on Al_2O_3	-	99.7	0	-	-
PtCo on Al_2O_3	-	326	-	0	-
AuCo on Al_2O_3	-	155.2 (193.8)	-	-	0 (0)
RuCo on CeO_2	6	48.6	-	-	-
PdCo aon CeO_2	-	61.2	0	-	-
PtCo on CeO_2	-	47.1	-	0	-
AuCo on CeO_2	-	54.2	-	-	0

Table S2: Results of the time resolved measurements of the cobalt leaching into the methanolic solution in the run with PtCo on Al_2O_3.

Time [h]	Co_ICP [µg/ml]
3	177
24	257
45	391

GC Method:

===
==

Agilent 8890

===
==

GC

GC Summary
Run Time 7.6 min
Post Run Time 0 min

Oven

Equilibration Time 0 min
Max Temperature 240 °C
(Initial) 40 °C
Hold Time 2 min
Post Run 110 °C
#1 Rate 25 °C/min
#1 Value 180 °C
#1 Hold Time 0 min

ALS

Front Injector

Syringe Size	10 µL
Injection Volume	1 µL
Solvent A Washes (PreInj)	5
Solvent A Washes (PostInj)	5
Solvent A Volume	8 µL
Sample Washes	1
Sample Wash Volume	8 µL
Sample Pumps	3
Solvent Wash Draw Speed	150 µL/min
Solvent Wash Dispense Speed	6000 µL/min
Sample Wash Draw Speed	150 µL/min
Sample Wash Dispense Speed	6000 µL/min
Injection Dispense Speed	6000 µL/min
Viscosity Delay	0 sec
L1 Airgap	0.2 µL

Front SS Inlet He

Mode	Split
Heater	On 180 °C
Pressure	On 0
Total Flow	On 133.4 mL/min
Septum Purge Flow	On 3 mL/min
Pre-Run Flow Test	Off
Gas Saver	On 20 After 2 min mL/min
Split Ratio	50 :1
Split Flow	127.84 mL/min
Liner	Agilent 5190-3165: 870 µL (Split. taper. wool. low pressure drop)

PolyArc

Temperature	
Setpoint	On
(Initial)	450 °C

Column #1

Column Information	Agilent 123-7033UI DB-WAX Ultra I
Temperature Range	20 °C—240 °C (240 °C)
Dimensions	30 m x 320 µm x 0.5 µm
In	Front SS Inlet He
Out	Aux EPC 1
(Initial)	40 °C
Pressure	0
Flow	2.5568 mL/min
Average Velocity	33.826 cm/sec
Holdup Time	1.4781 min
Control Mode	Constant Flow
(Initial)	2.5568 mL/min
Post Run	1 mL/min

Column #2
Column Information Agilent FS. Deactivate
Temperature Range 20 °C—240 °C (240 °C)
Dimensions 2.5 m x 250 µm x 0 µm
Out Front Detector FID
(Initial) 40 °C
Pressure 0
Flow 4 mL/min
Average Velocity 122.56 cm/sec
Holdup Time 0.033996 min
Control Mode Constant Flow
Setpoint On
(Initial) 4 mL/min
Post Run 9.5407 mL/min

Front Detector FID
Makeup He
Heater On 250 °C
H2 Flow On 1.5 mL/min
Air Flow On 350 mL/min
Makeup Flow On 25 mL/min
Carrier Gas Flow Correction Constant Makeup and Fuel FlowFlame
Initial Baseline Minimum 2 pA
Initial Baseline Maximum 20 pA
Initial Baseline Noise 0.3 pA
Final Baseline Minimum 2 pA
Final Baseline Maximum 40 pA
Final Baseline Noise 0.6 pA
Total Peak Area 100 pA*sec
Maximum Peak Height 3 pA
Time Window Start 0 min
Time Window End 0.5333333333 min

===
Column(s)
===

Column Description : DB-WAX Ultra I
Inventory# : autoID-2
Model# : 123-7033UI
Manufacturer : Agilent
Diameter : 320.0 µm
Length : 30.0 m
Film thickness : 0.50 µm
Void time : 1.478 min
Maximum Temperature: 240.0 °C

Comment :

Column Description : FS. Deactivate
Inventory# : autoID-3
Model# :
Manufacturer : Agilent
Diameter : 250.0 µm
Length : 2.5 m
Film thickness : 0.00 µm
Void time : 0.034 min
Maximum Temperature: 240.0 °C
Comment :

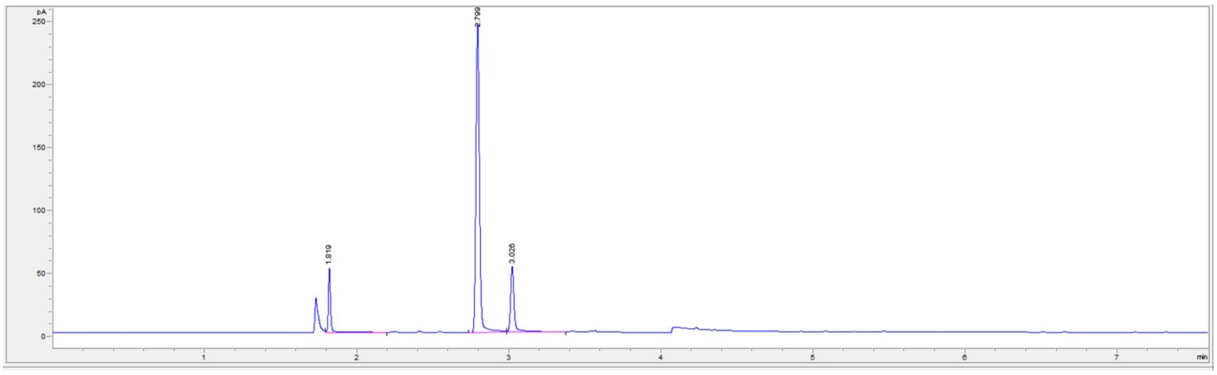

Figure S3: Exemplary chromatogram of a cobalt catalysed homologation reaction at 90 °C. RT 1.819 min = Dimethylether. RT 2.190 min = Acetaldehyde. RT 2.799 min = Acetaldehyde dimethylacetale. RT 3.026 min = Methylacetate.

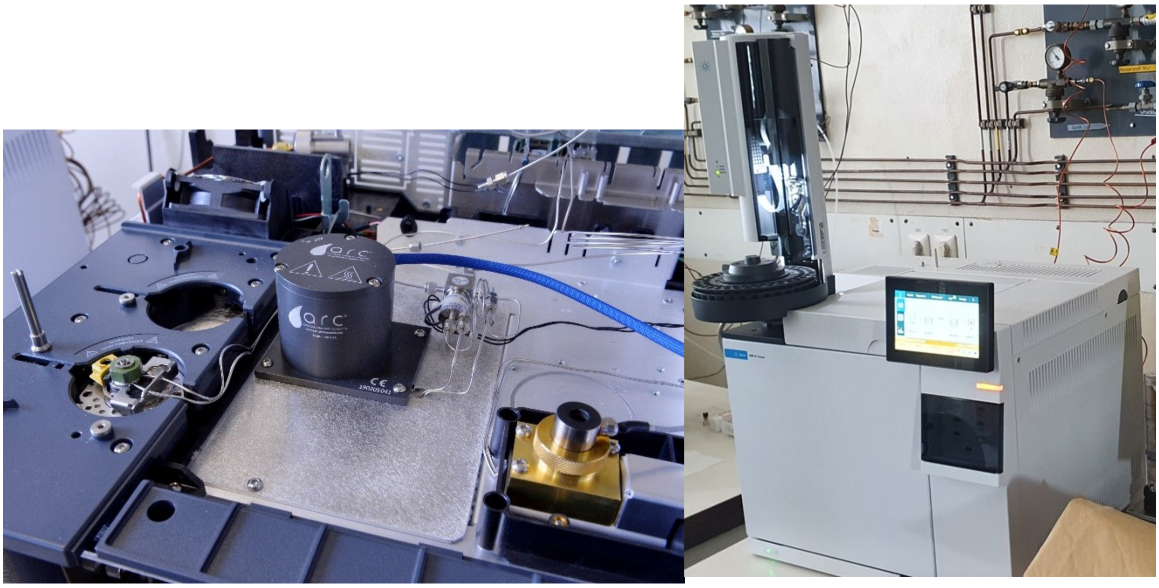

Figure S4: Picture of the gas chromatograph (right) and the PolyArc Reactor (left).

XRD-Diffractograms

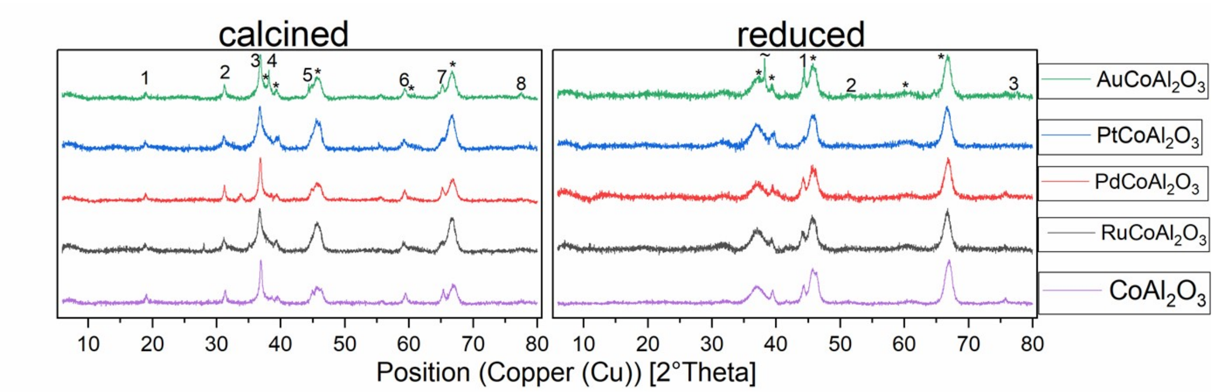

Figure S5: XRD-diffractograms of all tested heterogeneous catalysts on γ-Al_2O_3 in calcined and reduced state.

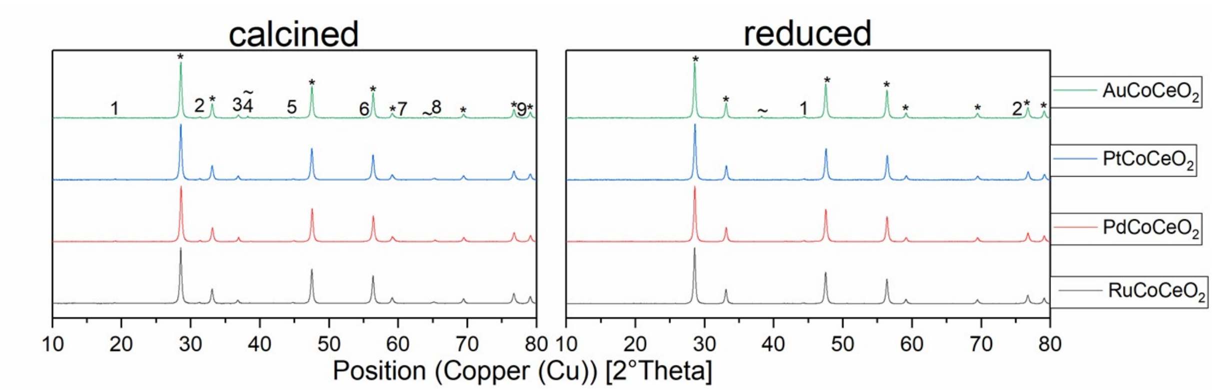

Figure S6: XRD-diffractograms of all tested heterogeneous catalysts on CeO_2 in calcined and reduced state.

Density functional theory

As a first step we compared DFT calculated and experimental enthalpies for the gas phase reactions and find good agreement:

$CO + 2\ CH_3OH$ — > CH_3COOCH_3 (Methylacetate) + H_2O — > ΔHexp= -142.6 kJ/mol
DFT = -140.736 kJ/mol

$CO + CH_3OH + H_2$ —> CH_3CHO (acetaldehyde) + H_2O — > ΔHexp= -96.5 kJ/mol
DFT = -99.456 kJ/mol

As a second step. we calculated reaction enthalpy for the hydrogenation reaction of dicobalt octacarbonyl to two cobalt tetracarbonyl hydrides $HCo(CO)_4$ for different GGA+U values (0. 4. 5. 6) and find the *respective values of* 36.2. 26.1. 24.4 *and* 22.6 *kJ/mol*. Calculated reaction enthalpy for U= 4 compares well with the experimental values provided in NIST Chemistry WebBook (13.4 - 27.6 kJ/mol) as well as the value obtained in the work in [Angew. Chem. 2014. 126. 8816 –8820].

In the calculation of the Gibbs free energy reaction diagrams. H_2. CO. CH_4 as well as all cobalt carbonyl species were treated as gas phase species while CH_3OH. H_2O. AA and MeOAc as liquid phase by using S values for the liquid phase and by correcting enthalpy value for the liquid-gas difference obtained from the NIST Chemistry WebBook. Total energies. zero point energy correction energies. cpdT and S values are provided in the Table S1 in the SI.

All of the Figures discussed in the DFT part of the main text can be found here. The Figure 4 of the main text was separated into three different Figures for better understanding. these separated Figures are shown in the supporting information Figures S10-S12.

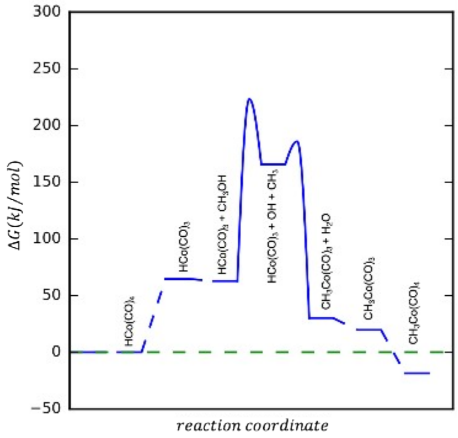

Figure S7: Reaction energy diagram for the CH_3OH activation and $CH_3Co(CO)_3$ formation on $HCo(CO)_4$ at T= 423.15 K

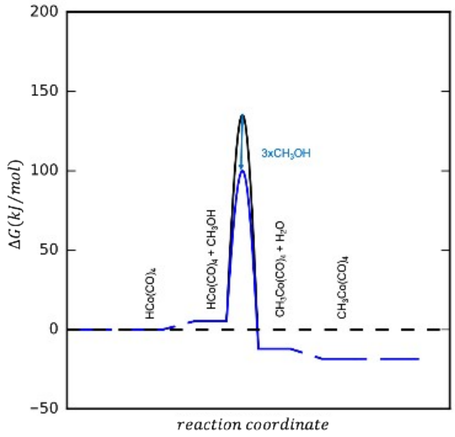

Figure S8: Reaction energy diagram for the CH_3OH protonation and $CH_3Co(CO)_3$ formation on $HCo(CO)_4$ at T= 423.15 K. Blue arrow shows the reduction of the barrier by increasing the number of methanol molecules.

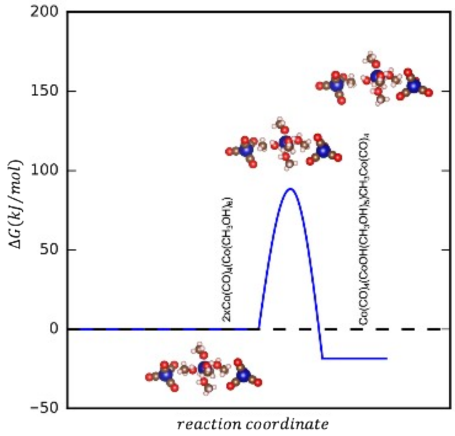

Figure S9: Reaction energy diagram for the disproportionation start reaction in $2xCo(CO)_4[Co(CH_3OH)_6]$.

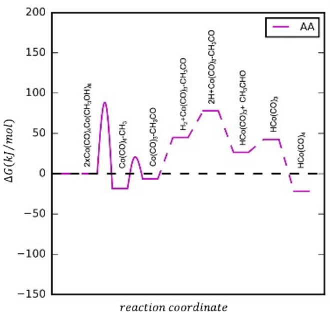

Figure S10: Reaction energy diagram for AA formation on $HCo(CO)_4$ at T= 423.15 K. p(CO)=20 bar. p(H_2) = 60 bar.

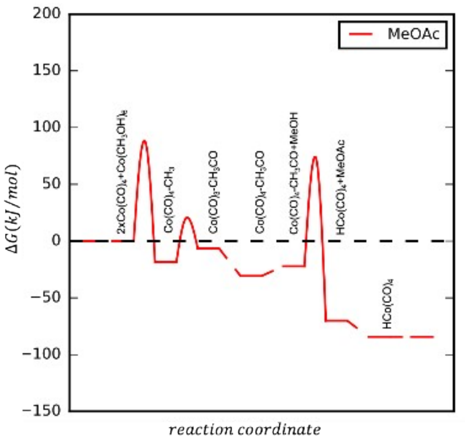

Figure S11: Reaction energy diagram for MeOAc formation on $HCo(CO)_4$ at T= 423.15 K. p(CO)=20 bar. p(H_2) = 60 bar.

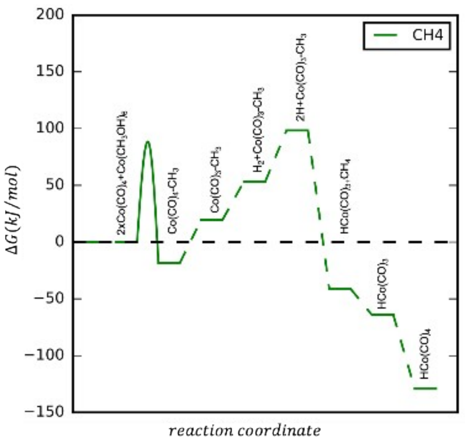

Figure S12: Reaction energy diagram for CH_4 formation on $HCo(CO)_4$ at T= 423.15 K. p(CO)=20 bar. p(H_2) = 60 bar.

Table S3: DFT calculated total energies (eV). zero point energy (eV). cpdT (eV) and S (eV/T) values.

	E	ZPE	cpdT	S
CO(g)	-12.07474556	0.137	0.108	0.002117
H_2O(l)	-12.80978117<br>-0.2846	0.582	0.122	0.00072865
H_2(g)	-7.16240358	0.279	0.125	0.001454
CH_3OH(l)	-27.74244005<br>-0.2648	1.380	0.172	0.001325

AcH(l)	-35.40713962<br>-0.119	1.483	0.172	0.00122188
MeOAc(l)	-56.28467272<br>-0.218	2.382	0.277	0.00270208
CH_4(g)	-23.275	1.205	0.183	0.0023567
$HCo(CO)_4$	-54.86862657	1.054	0.513	0.0051078
$HCo(CO)_4+CH_3OH$	-82.73115255	2.450	0.755	0.0068202
TS	-81.206	2.36	0.812	0.0071504
$Co(CO)_4-CH_3+H_2O$	-83.0172	2.474	0.785	0.0067006
$Co(CO)_4-CH_3$	-70.07488619	1.860	0.602	0.0055474
TS	-69.6516	1.848		
$Co(CO)_3-CH_3CO$	-69.99392876	1.884	0.584	0.0054584
$H_2-Co(CO)_3-CH_3CO$	-77.06857435	2.320	0.645	0.0057215
$2H-Co(CO)_3-CH_3CO$	-76.6947	2.302	0.636	0.0057297
TS	-76.62	2.302	0.682	0.0059472
$HCo(CO)_3-CH_3CHO$	-77.13440433	2.323	0.660	0.0060621
$HCo(CO)_3$	-41.28142491	0.712	0.469	0.0048734
$HCo(CO)_3-CH_3OH$	-69.66847049	2.269	0.610	0.0056881
TS	-67.93955367	2.24	0.596	0.0054381
$OH-HCo(CO)_3-CH_3$	-68.586112	2.200	0.618	0.0055514
TS	-68.27952103	2.162	0.638	0.0057518
$Co(CO)_3-CH_3+H_2O$	-70.0013	2.274	0.646	0.0058027
$Co(CO)_3-CH_3$	-56.96434718	1.620	0.496	0.0049584
$Co(CO)_3-CH_3+H_2$	-64.23331176	2.049	0.557	0.0051874
$Co(CO)_3-CH_3+2H$	-63.77965687	2.054	0.535	0.0051063
TS	-63.66973241	1.97	0.567	0.0054314
$HCo(CO)_3-CH_4$	-64.91091259	2.042	0.621	0.0060442
$Co(CO)_4-CH_3CO$	-82.86346751	2.098	0.711	0.0062634
$Co(CO)_4-CH_3CO+CH_3OH$	-110.8507837	3.50	0.932	0.007608
TS	-109.98302	3.45	0.748	0.0069630
$HCo(CO)_4$-MeOAc	-111.3323544	3.472	0.899	0.0074895
